# Physiological and biochemical evaluation of high anthocyanin pigmented tea (*Camellia sinensis* L. O. Kuntze) germplasm for purple tea production

**DOI:** 10.3389/fnut.2022.990529

**Published:** 2022-08-31

**Authors:** Pradeep Kumar Patel, Shahida Anusha Siddiqui, Kamil Kuča, Santanu Sabhapondit, Rupak Sarma, Boby Gogoi, Shobhit Kumar Singh, Ranjeet Kumar Bordoloi, Jayanta Kumar Saikia, Romen Chandra Gogoi, Kanchan Bhardwaj, Jie Yang, Yang Tao, Sivakumar Manickam, Buddhadeb Das

**Affiliations:** ^1^Department of Plant Physiology and Breeding, Tocklai Tea Research Institute, Tea Research Association, Jorhat, India; ^2^Campus Straubing for Biotechnology and Sustainability, Technical University of Munich, Straubing, Germany; ^3^German Institute of Food Technologies (DIL eV), Quakenbrück, Germany; ^4^Department of Chemistry, Faculty of Science, University of Hradec Kralove, Hradec Kralove, Czechia; ^5^Biomedical Research Center, University Hospital Hradec Kralove, Hradec Kralove, Czechia; ^6^Department of Biochemistry, Tocklai Tea Research Institute, Tea Research Association, Jorhat, India; ^7^Tea Testing Laboratory, Tocklai Tea Research Institute, Tea Research Association, Jorhat, India; ^8^School of Biological and Environmental Sciences, Shoolini University of Biotechnology and Management Sciences, Solan, India; ^9^Jiangsu Key Laboratory of Marine Bioresources and Environment/Jiangsu Key Laboratory of Marine Biotechnology, Jiangsu Ocean University, Lianyungang, China; ^10^College of Food Science and Technology, Nanjing Agricultural University, Nanjing, China; ^11^Petroleum and Chemical Engineering Department, Faculty of Engineering, Universiti Teknologi Brunei, Bandar Seri Begawan, Brunei; ^12^North Bengal Regional R&D Centre, Tea Research Association, Nagrakata, India

**Keywords:** purple tea, anthocyanin, carotenoids, cleft grafting, germplasm, diversification, net photosynthesis

## Abstract

Finding promising purple tea germplasm that would target new tea products for diversification and value addition boost the tea industry’s economic growth. Accordingly, 10 tea germplasm viz. TRA St. 817, TRA St. 293, TRA St. 400, TRA 177/3, TRA 376/2, TRA 376/3, TRA 427/7, TRA P7, TRA P8, and TV1 were evaluated in terms of gas exchange parameters, multiplication performance, and biochemical markers such as chlorophyll, carotenoids, and anthocyanin content, which are related to the purple tea quality. The investigated gas exchange and biochemical parameters revealed significant differences. Germplasm TRA St.817 was physiologically more efficient (24.7 μmol m^–2^ s^–1^), followed by TRA St. 293, exhibiting the highest net photosynthesis, water use efficiency (19.02 μmol mmol^–1^), carboxylation efficiency (0.73), chlorophyll fluorescence or photochemical efficiency of PSII (0.754) and mesophyll efficiency (ci/gs ratio: 2.54). Net photosynthesis was positively correlated with water use efficiency, carboxylation efficiency, mesophyll efficiency, and photochemical efficiency of PSII (*r* = 0.965^**^, 0.937^**^, 0.857^**^, 0.867^**^; *P* = 0.05), respectively, but negatively correlated with the transpiration ratio (*r* = −0.878^**^; *P* = 0.05) based on Pearson correlation analysis. The total anthocyanin content (4764.19 μg.g^–1^ fresh leaf weight) and carotenoid content (3.825 mg.g^–1^ fresh leaf weight) were highest in the TRA St.817 germplasm, followed by germplasm TRA St. 293 (2926.18 μg.g^–1^ FW). In contrast, total chlorophyll content was significantly low (1.779 mg.g^–1^ fresh weight), which is very suitable for manufacturing purple tea. The highest carotenoid concentration in TRA St. 817 was 3.825 mg.g^–1^ FW, followed by TRA P8 (3.475 mg.g^–1^ FW), favoring the formation of more volatile flavor constituents. The promising germplasm, TRA St 817, has a multiplication success rate of 91.4% through cleft grafting. The outcome reveals that TRA St.817 is a promising germplasm that can be used to make speciality teas, i.e., purple tea.

## Introduction

Tea (*Camellia sinensis* L. O. Kuntze) is the world’s oldest beverage, second to water (1–3). Purple tea is now becoming widely attractive in domestic and global markets regarding health benefits. Robert Bruce began producing tea in the Indian state of Assam in 1823. The National Active Germplasm Site (NAGS) of TTRI is the world’s largest tea germplasm collection center (4). It has released 35 TV clones, 153 TRA/garden series clones, and 16 bi-clonal hybrid seed stocks and made genetic resources available to other tea-producing countries (5).

Anthocyanins pigments are found in red grapes (6, 7), berries (8), eggplant (9–11), and sweet potatoes (12). They demonstrate pH-dependent colors (13), contributing to the appealing colors of fruits, vegetables, and flowers (6, 14, 15) and accumulate in the vacuoles (16, 17). They have gained popularity due to their potential health benefits (6, 18), including antioxidant (19), anti-carcinogenic (20), anti-angiogenic (21, 22), antimicrobial (23–26), atherosclerosis (24), anti-apoptotic (27), and pro-apoptotic (12, 28) properties. Plants are susceptible to UV-B irradiation due to their sedentary nature, causing oxidative stress. When plants are exposed to UV-B irradiation, their biosynthesis is up-regulated (29, 30) and protected by anthocyanins. Besides protecting against pests and herbivores, leaf temperature during winter (31, 32) and the production of red wine (7, 33, 34) are controlled.

The newly discovered tea germplasm was not studied in terms of physiological, biochemical, quality evaluation, and multiplication efficiency, even though it has a higher amount of anthocyanins. The impact of anthocyanins in this novel germplasm on catechin levels was also determined as they are products of the shikimate and phenylpropanoid pathways. In addition to catechins, anthocyanins are expected to contribute to developing new and unique tea products. This study aims to understand the 10 selected germplasm’s physiological, biochemical, and quality variations. In this direction, the key objectives of the present investigation are (i) Identification of high anthocyanin pigmentation tea germplasm from existing tea plantation areas, (ii) Physiological, biochemical, and qualitative evaluation of identified purple tea germplasm to produce a speciality tea, i.e., anthocyanin-rich tea (purple tea), and (iii) To meet the demand for purple tea production on a commercial scale, as well as an investigation into the multiplication efficiency of the promising purple tea germplasm. Also, in terms of importance and scope, this study identifies the promising purple tea germplasm for the first time that could be utilized to diversify and enhance new tea products, consequently boosting tea productivity and promoting the economic growth of the tea industry.

## Materials and methods

A total of 10 tea germplasms [*Camellia sinensis* (L.) O. Kuntze] was used for the study. Out of 10, nine pigmented purple tea germplasms, viz. TRA St. 817, TRA St. 293, TRA St. 400, TRA 177/3, TRA 376/2, TRA 376/3, TRA 427/7, TRA P7, TRA P8, and one non-pigmented tea germplasm TV1 were selected from the core collection available at TTRI germplasm bank (Photo plate I). The experimental soil was sandy, with a pH of 4.5–5.0. Photosynthesis, chlorophyll fluorescence (fv/fm), chlorophyll and carotenoids were observed on the maintenance leaves (physiologically more active leaves) at the plucking table between 9:30 and 11:30 a.m. For multiplication through cleft grafting, TV18 rootstocks were used.

### Gas exchange parameters

Gas exchange parameters were measured by Portable Photosynthetic System (CIRAS-II, PP System, Amesbury, MA, United States).

### Chlorophyll fluorescence

Chlorophyll fluorescence parameters were measured by a pulse amplitude modulation chlorophyll fluorometer JUNIOR-PAM (Walz, Effeltrich, Germany).

### Extraction, identification and quantification of anthocyanins

#### Extraction

5 g of grounded leaf sample were weighed into a 250 ml conical flask covered with a foil and mixed with 50 ml CH_3_OH/HCl (99:1 v/v) and magnetically stirred at 900 rpm for 4 h at room temperature. The resultant solution was filtered and evaporated to dryness at room temperature (<35°C). The extract was dissolved in 10 ml distilled water, passed through a 0.45 μm membrane filter, and kept in an ice bath for analysis (35).

The extracts were passed through a reverse phase (RP) C_18_ solid phase extraction (SUPELCO, SPE, Sigma-Aldrich, United States) cartridges previously activated with 10% CH_3_OH/HCl (HPLC grade, Sisco Research Laboratories Pvt. Ltd., Mumbai, India). Anthocyanins were adsorbed in the cartridge while sugars, acids and other water-soluble compounds were washed out using 0.01% HCl. Anthocyanins were then recovered using acidified methanol (10% Formic acid v/v). The cartridges were washed with ethyl acetate (Thermo Fischer Scientific, United Kingdom) to remove phenolic compounds retained in the cartridge. The purified extracts were stored at 10°C until further analysis.

#### Identification and quantification of anthocyanins

The anthocyanin extracts from the samples were analyzed for anthocyanins profile using HPLC (Agilent 1260 LC System, 5301 Stevens Creek Blvd Santa Clara, CA 95051, United States). A Gemini C_18_ ODS column was used for HPLC analysis, and the detector was set at 520 nm (36). An injection volume of 20 μl with a 1 ml/min flow rate and the oven temperature of 35°C were used. The eluents were mobile phase A (water/acetonitrile/formic acid, 87/3/10 v/v/v, FINAR Ltd./Sisco Research Laboratories Pvt. Ltd., Mumbai, India) and mobile phase B (100% HPLC grade Acetonitrile, Sisco Research Laboratories Pvt. Ltd.). The chromatographic conditions were: 3% B in A at the time of injection at 45 min; 25% B in A at 46 min; 30% B in A at 47 min; 3% B in A (initial conditions). The anthocyanin standards *Cyanidin-3-O-glucoside, Cyanidin-3-O-galactoside, Cyanidin chloride, Peonidin chloride, Pelargonidin chloride, and Malvidin chloride* were obtained from Sigma Aldrich, United Kingdom, to identify and quantify anthocyanin fractions.

### Determination of caffeine and catechins

Caffeine and individual catechin content in tea samples were quantified following ISO 14502-2 (37). Fresh leaves were enzyme denatured, oven dried, and finely powdered. 0.2 g of the powdered material was accurately weighed and extracted with 5 ml of 70% aqueous methanol and refluxed in a water bath at 70°C for 10 min. After cooling, the extracts were centrifuged at 3,000 rpm for 10 min. The supernatant was transferred into a 10 ml volumetric flask. The process was repeated with another 5 ml extraction solvent. 1 ml of the extract was diluted with a freshly prepared stabilizing agent five times. The stabilizing agent was prepared from10% (volume fraction) acetonitrile with 500 μg/ml of EDTA and ascorbic acid (Sisco Research Laboratories Pvt. Ltd., Mumbai, India). This diluted extract was filtered through 0.45 μm syringe filters before quantitative estimation using High-Performance Liquid Chromatography. The estimation was carried out using UPLC (Dionex, Ultimate 3000) with Luna 5 μm phenylhexyl Phenomenex column (4.5 mm × 250 mm; Torrance, CA, United States) and UV-Vis detector set at 278 nm. The column temperature was set at 25 ± 0.5°C. Mobile phase A consisted of 2% (v/v) acetic acid (Sisco Research Laboratories Pvt. Ltd., Mumbai, India), 9% (v/v) acetonitrile and ultrapure water. Mobile phase B consisted of 80% (v/v) acetonitrile and ultrapure water. The injection volume was 10 μl, and the flow rate was 1 ml/min. The gradient elution was set as 100% mobile phase A for 10 min, then a linear gradient to 68% mobile phase A, 32% mobile phase B over 15 min and held at this composition for 10 min. Catechin and caffeine peaks were identified by comparing retention times from sample chromatograms with those obtained from the standard solutions of catechins under the same chromatographic conditions. Estimating individual catechins and caffeine was done using Relative Response Factors of catechins concerning caffeine, as described in ISO 14502-2 (37).

### Methodology of preparation of tea infusion and liquor for organoleptic evaluation

#### Tea infusion

The methodology for preparing tea infusion was as per (38) E. Soluble substances from a dried tea leaf were extracted in porcelain using fresh boiling water, and the liquor was poured into white porcelain. The organoleptic qualities of the infused leaf and the liquid without milk were evaluated. For liquor preparation, 2.8 ± 0.2 g of tea was transferred to the pot and weighed. The teapot was filled with fresh boiling water within 4–6 mm of the brim and covered with a lid. To brew tea, 4–5 min was allowed. The liquor was poured into the bowl through the serration, using the cover to keep the infused leaf in the pot. The lid was removed and then inverted. To inspect the infused leaf, it was placed on the inverted lid.

#### Liquor

To make a cup of tea, 2.8 ± 0.2 g of tea was weighed into a 150 ml cup, added with freshly boiled water, steeped for 5 min, and filtered. The infused leaves were pressed into the sampling pot’s lid. A randomized technique was followed for the tasting by the tea taster, where the sample details were hidden following ISO 3163 (39). A tea taster slurps the drink into his or her mouth noisily while using a large spoon once it has cooled sufficiently. This ensures that the tea and plenty of oxygen pass through the tongue’s taste receptors for a consistent taste profile (40). Before the next sample, the liquid was spat into a spittoon. FAO (41) describes the tea quality parameters (liquor color, strength, briskness, and brightness), manufacturing flaw parameters, and field-related agro-practices. The tea quality was rated out of 10 once the taster considered these observations (42).

### Statistical analysis

All the determinations were carried out in triplicate, and values were expressed as the mean ± SEm. Statistical significance was examined through a one-way analysis of variance and Duncan’s test at *p* ≤ 0.05 using the Statistical Package WASP 2.0.

## Results and discussion

### Gas exchange parameters

**FIGURE 1:**
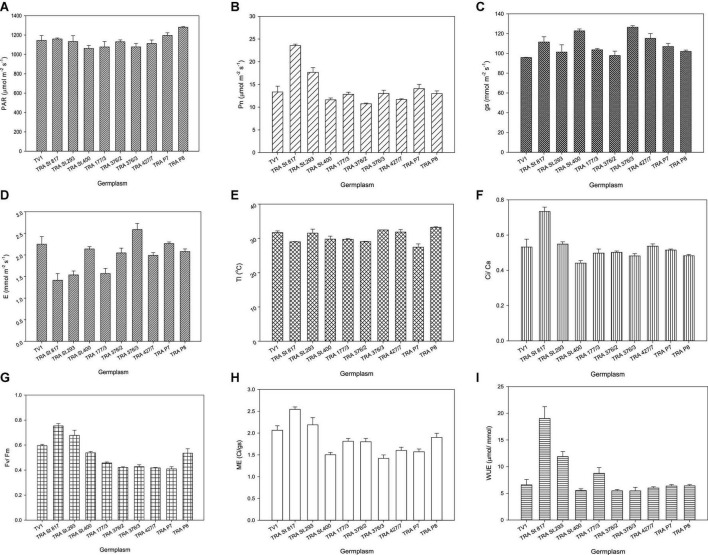
Physiological responses of tea germplasm with respect to **(A)** light (PAR), **(B)** net photosynthesis (Pn), **(C)** stomatal conductance (Gs), **(D)** evaporation (E), **(E)** leaf temperature (Lt), **(F)** carboxylation efficiency (ci/ca), **(G)** chlorophyll fluorescence (fv/fm), **(H)** mesophyll efficiency (ME), **(I)** water-use efficiency (WUE). Vertical bars indicate the standard error of means.

PAR varied from 1,062 to 1,281 μmol m^–2^ s^–1^ (Figure 1A). Net photosynthesis and other growth determining factors of selected germplasm, viz. TRA St. 817, TRA St. 293, TRA St. 400, TRA 177/3, TRA 376/2, TRA 376/3, TRA 427/7, TRA P7, and TRA P8 and TV1 depend upon the efficient use of light for assimilating CO_2_. In Northeast India, Pn reduction begins when light intensity exceeds 1,200 μmol m^–2^ s^–1^ (43, 44). Pn varies significantly among the germplasm, and the highest Pn was recorded in TRA St. 817 (24.7 μmol m^–2^ s^–1^), followed by TRA St. 293 (21.3 μmol m^–2^ s^–1^). In contrast, the lowest Pn was noticed in TRA St. 400 (10.4 μmol m^–2^ s^–1^) (Figure 1B). Barman et al. (44) correlated Pn with PAR (maximum Pn of 13.95 μmol m^–2^ s^–1^ at the PAR of 1,200 μmol m^–2^ s^–1^), whereas Hajara and Kumar (45, 46) recorded the maximum Pn at 11.9 μmol m^–2^ s^–1^ at the highest PAR (1,370 μmol m^–2^ s^–1^). Leaf temperature plays a crucial role in the tea leaf photosynthesis process. Leaf temperatures also showed significant variations among germplasm. TRA P8 exhibited a maximum leaf temperature of 33.3°C. Its corresponding Pn was 13.0 μmol m^–2^ s^–1^, whereas, at 29°C, maximum Pn was observed in TRA St. 817 (24.7 μmol m^–2^ s^–1^) (Figure 1C). The maximum Pn tea leaf temperature required is usually in the range of 25–30°C (47), which declines above 35°C resulting in no Pn above 39°C (48).

Whitehead et al. (49) noticed that PAR influences stomatal conductance, and Lt. E substantially limits the Gs (50, 51). The highest Gs was 122.8 mmol m^–2^ s^–1^, and the corresponding E was 2.14 mmol m^–2^ s^–1^, whereas the lowest Gs was 95.8 mmol m^–2^ s^–1^, and its corresponding E was 2.24 mmol m^–2^ s^–1^. Among the germplasm, TRA St. 400 presented the highest Gs (122.8 mmol m^–2^ s^–1^). In contrast, the TV1 showed the lowest Gs (95.8 mmol m^–2^ s^–1^), and the corresponding E was 2.14 and 2.24 mmol m^–2^ s^–1^, respectively (Figures 1D,E), indicating a positive correlation between gs and E.

The highest WUE was observed in TRA St. 817 (19.02 μmol mmol^–1^), followed by TRA St. 293 (11.87 μmol mmol^–1^) (Figure 1I). C_*i*_/C_a_ was found within 0.44–0.73, similar to other C_3_ plants (52–54). Higher WUE is associated with a higher Pn/E ratio (55, 56). Maximum Ci/Ca was observed in TRA St. 817 (0.73) (Figure 1F), whereas the minimum Ci/Ca was noticed in the non-anthocyanin pigmented clone TV1 (0.44). Pn is linked to Gs and is substrate limited. The present study suggests that the mesophyll factor also plays an important role in Pn regulation through its effect on determining the CO_2_ concentration gradient. Higher Ci/Ca ascertains a better mesophyll factor, i.e., ME. Germplasm TRA St. 817 (2.54) showed maximum ME followed by TRA St. 293 (2.19) (Figure 1H).

Chlorophyll fluorescence (fv/fm) reflects the maximal efficiency of excitation energy captured by “open” PSII reaction centers. fv/fm showed significant variations among the germplasm. The highest fv/fm was observed in TRA St. 817 (0.754), followed by TRA St. 293 (0.678) (Figure 1G), whereas the lowest was noticed in TRA P7 germplasm. A decreased fv/fm indicates a down-regulation of photosynthesis or photoinhibition (57–59). Germplasm TRA St. 817 is more physiologically efficient than other pigmented and non-pigmented germplasm.

#### Correlation of physiological parameters in the selected germplasm

**FIGURE 2:**
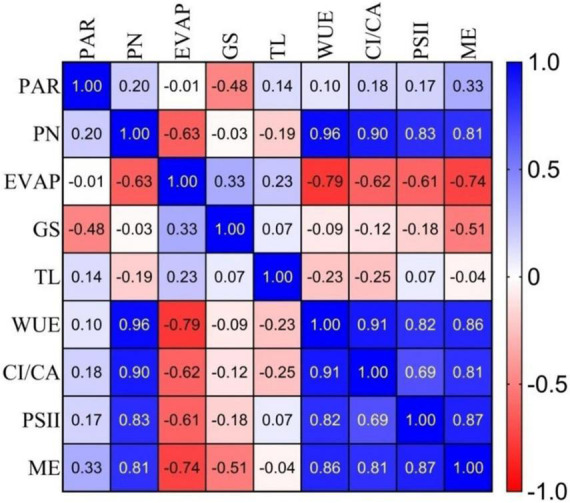
Correlation coefficients (r) among gas exchange, photochemical efficiency of PSII and mesophyll efficiency parameters of selected germplasm. Where: PAR, Photosynthetic Active Radiation; PN, Net Photosynthesis; EVAP, Transpiration; GS, Stomatal Conductance; TL, leaf temperature; WUE, Water Use Efficiency; CI/CA, Carboxylation efficiency; PSII, Photochemical efficiency of PSII; and ME, Mesophyll efficiency.

The Pearson correlation coefficient among the major group of anthocyanin pigments for the 10 genotypes was calculated and presented in Figure 2. Net photosynthesis showed a high positive correlation with WUE (*r* = 0.96), Ci/Ca (*r* = 0.90), PSII or fv/fm (*r* = 0.83), and ME (*r* = 0.81) in the selected anthocyanin pigmented tea germplasm for the studied purple tea (Figure 2).

### Pigment profiles

The anthocyanidins fractions were identified and quantified by HPLC from the purple-colored tea clones using pure anthocyanin standards. The anthocyanins content of the selected germplasm was from 988.62 to 4764.19 μg g^–1^ FW (Table 1). The germplasm TRA St. 817 exhibited the highest total anthocyanin content (4764.19 μg g^–1^ FW), followed by germplasm TRA St. 293 (2926.18 μg g^–1^ FW). Malvidin is the most predominant form of the purple-colored tea clones, similar to Bilberry (*Vaccinium myrtillus*) with malvidin-3-glucoside (60, 61); blueberry with malvidin-3-arabinoside, malvidin-3-glucoside and malvidin-3-galactoside (62); red grapes with malvidin-3-glucoside (63, 64). All edible berries contain malvidin-3-glucoside, malvidin-3-glucoside acetate, and malvidin-3-glucoside coumarate (65). Products like young red wine (7, 66), Italian red wine (34, 64), and grape juice (65) also contain malvidin as the predominant anthocyanin. It is a positive step toward the diversification of tea products and uses of tea.

**TABLE 1:** Anthocyanidin concentrations (μgg^–1^ fresh weight) in green leaf and purple leaf colored clones.

Selections	Cyanidin 3-O- glucoside	Cyanidin-3-O-galactoside	Peonidin chloride	Cyanidin chloride	Pelargonidin chloride	Malvidin chloride	Total anthocyanin
***Green leaf colored***							
TV1	−	−	−	−	−	−	−
***Purple leaf colored***							
TRA St 817	222.64 ± 38.46^a^	82.98 ± 11.66^a^	12.06 ± 0.69^ab^	1650.86 ± 555.10^a^	912.80 ± 184.34^ab^	1911.30 ± 424.16^a^	4792.64 ± 80.89^a^
TRA St.293	27.01 ± 0.74^b^	527.52 ± 39.02^a^	8.46 ± 1.48^bc^	141.07 ± 31.09^a^	1075.92 ± 112.49^a^	1146.20 ± 220.16^a^	2926.18 ± 403.50^b^
TRA St.400	15.40 ± 4.93^b^	34.78 ± 13.87^a^	4.84 ± 0.15^cd^	134.65 ± 2.13^a^	369.90 ± 60.22^bc^	429.05 ± 69.33^a^	988.62 ± 136.21^cd^
TRA 177/3	52.57 ± 14.99^b^	202.28 ± 24.8^a^	7.82 ± 0.37^bc^	121.08 ± 18.16^a^	351.15 ± 11.41^c^	945.17 ± 297.09^a^	1680.08 ± 344.01^bcd^
TRA 376/2	22.82 ± 0.46^b^	254.89 ± 44.42^a^	6.18 ± 0.38^c^	124.49 ± 1.75^a^	280.22 ± 19.87^c^	901.83 ± 24.83^a^	1590.43 ± 46.78^b*cd*^
TRA 376/3	17.40 ± 1.23^b^	275.52 ± 4.78^a^	4.85 ± 0.53^cd^	168.62 ± 0.78^a^	148.45 ± 16.50^c^	664.86 ± 66.91^a^	1279.69 ± 77.16^bcd^
TRA 427/7	14.61 ± 0.12^b^	81.78 ± 27.37^a^	7.87 ± 0.04^bc^	86.01 ± 9.27^a^	169.00 ± 4.63^c^	794.35 ± 62.41^a^	1153.62 ± 39.58^cd^
TRA P7	15.09 ± 0.12^b^	360.27 ± 113.67^a^	14.82 ± 0.72^a^	86.97 ± 19.63^a^	81.39 ± 12.72^c^	1330.63 ± 156.48^a^	1889.18 ± 74.32^cd^
TRA P8	4.17 ± 0.18^b^	164.88 ± 58.76^a^	8.20 ± 1.48^bc^	221.22 ± 41.44^a^	123.18 ± 49.14^c^	1123.72 ± 393.10^a^	1645.38 ± 461.22^d^

Values are Mean ± Standard error of the mean (SEm). Different parameters were analyzed using ANOVA to detect a significant difference between means. Means were compared using Duncan’s Multiple Range Test (DMRT) at *P* < 0.05. Mean of the same alphabets in columns are not significant.

#### Correlation of major group of anthocyanin pigments in the selected germplasm

**FIGURE 3:**
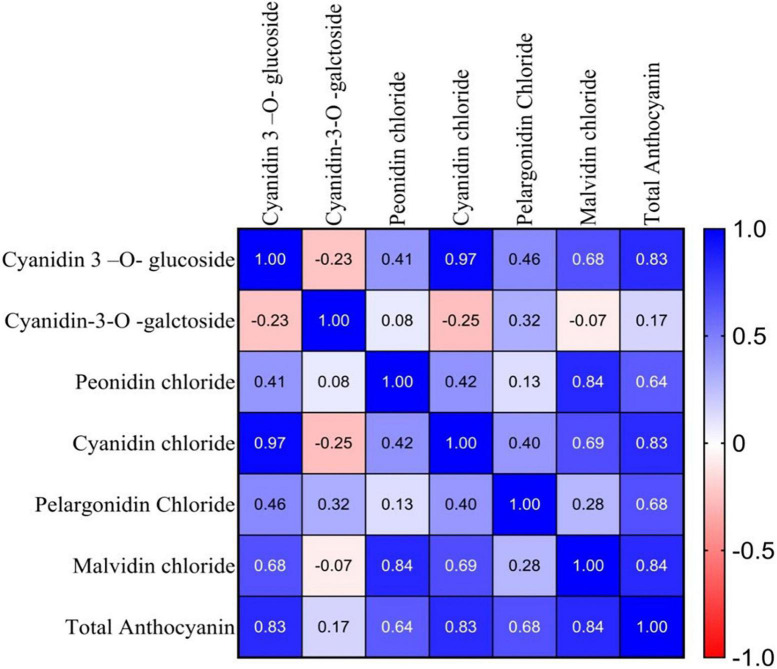
Correlation coefficients (r) between major groups of anthocyanin pigments of selected germplasm.

The Pearson correlation coefficient among the major group of anthocyanin pigments for the 10 genotypes was calculated and presented in Figure 3. Total anthocyanin content showed a high positive correlation with cyanidin 3-o-glucoside (*r* = 0.83), peonidin chloride (*r* = 0.64), cyanidin chloride (*r* = 0.83) pelargonidin chloride (*r* = 0.68), and malvidin chloride (*r* = 0.84), in the selected anthocyanin pigmented tea germplasm for the studied purple tea (Figure 3).

### Catechins and caffeine

Besides anthocyanin, a comparison of catechins and caffeine content of the selected germplasm was attempted. The catechin and caffeine contents of the standard non-pigmented (green-colored) quality cultivar (TV1) and purple-colored tea germplasm were determined by HPLC using pure catechin standards. Catechins and caffeine normally present in the green-leaf colored tea plants were also present in the purple-colored tea cultivars, namely, EGC, + C, EC, EGCG, and EGC (Table 2). The highest total catechin content was recorded in TRA P8 (17.38%) and TRA P7 (16.34%), followed by TRA 376/2 (14.07%), whereas the caffeine content was noted in TRA P8 (1.46%), TRA P7 (0.59%), and TRA St. 817 (2.17%). A representative HPLC chromatogram is shown in Supplementary Figure 1.

**TABLE 2:** Catechin and caffeine content (%) in green and purple colored leaves of tea germplasm.

Selections	Caffeine	EGC	+ C	EC	EGCG	ECG	Total catechin
TV1	4.59 ± 0.12^ab^	2.46 ± 0.05^bc^	0.70 ± 0.05^b^	1.31 ± 0.03^cd^	7.23 ± 0.36^c^	4.60 ± 0.02^a^	16.28 ± 0.50^b^
TRA St 817	2.17 ± 0.29^de^	1.18 ± 0.05^d^	0.39 ± 0.02^bc^	0.19 ± 0.01^f^	5.71 ± 0.16^d^	0.30 ± 0.01^h^	7.77 ± 0.23^f^
TRA St.293	3.36 ± 0.13^c^	2.68 ± 0.09^abc^	ND	1.55 ± 0.12^bc^	4.01 ± 0.68^e^	2.37 ± 0.08^c^	10.61 ± 0.39^d^
TRA St.400	3.63 ± 0.49^c^	2.68 ± 0.21^abc^	1.10 ± 0.33^a^	1.31 ± 0.05^cd^	4.81 ± 0.16^de^	3.82 ± 0.06^b^	13.72 ± 0.17^c^
TRA 177/3	3.02 ± 0.32^cd^	3.51 ± 0.71^a^	0.08 ± 0.01^cd^	1.29 ± 0.04^cd^	6.90 ± 0.32^c^	2.10 ± 0.01^d^	13.88 ± 0.42^c^
TRA 376/2	4.81 ± 0.31^a^	2.78 ± 0.10^ab^	ND	1.13 ± 0.01^d*e*^	8.48 ± 0.03^b^	1.68 ± 0.01^f^	14.07 ± 0.13^c^
TRA 376/3	2.32 ± 0.79^de^	2.16 ± 0.40^bcd^	0.08 ± 0.01^cd^	0.88 ± 0.13^cd^	4.91 ± 0.10^de^	1.52 ± 0.04^g^	9.55 ± 0.34^e^
TRA 427/7	3.79 ± 0.30^bc^	2.74 ± 0.08^ab^	ND	1.40 ± 0.04*^e^*	7.03 ± 0.17^c^	1.84 ± 0.08^e^	13.01 ± 0.21^c^
TRA P7	0.59 ± 0.04^f^	1.71 ± 0.05^cd^	0.19 ± 0.07^cd^	1.87 ± 0.01^ab^	12.39 ± 0.41^a^	0.19 ± 0.03^h^	16.34 ± 0.43^b^
TRA P8	1.46 ± 0.04^ef^	2.08 ± 0.01^bcd^	0.15 ± 0.03^cd^	2.16 ± 0.27^a^	12.69 ± 0.14^a^	0.31 ± 0.02^h^	17.38 ± 0.14^a^

Values are Mean ± Standard error (SEm). Different parameters were analyzed using ANOVA to detect a significant difference between means. Means were compared using Duncan’s Multiple Range Test (DMRT) at *P* < 0.05. Mean of the same alphabets in columns are not significant. ND-Not detected.

The values of EGC, + C, EC, EGCG, ECG, and Total Catechin are on a% dry mass basis.

EGC, Epigallo catechin; + C, Catechin; EC, Epicatechin; EGCG, Epigallo catechin gallate; ECG, Epicatechin gallate.

ND, Not detected.

### Chlorophyll and carotenoids

**FIGURE 4:**
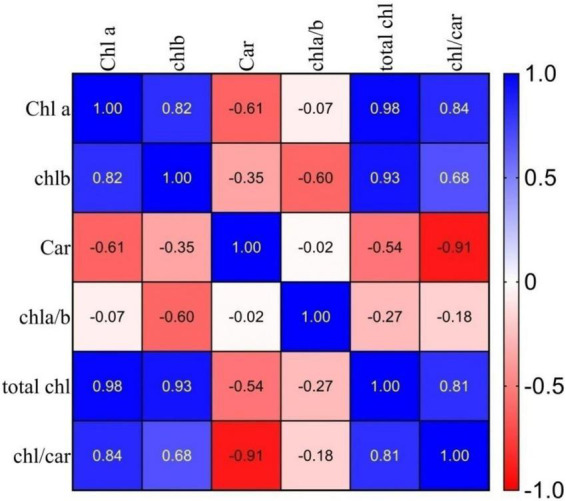
Correlation coefficients (r) between chlorophyll and carotenoid pigments of selected germplasm. Where: Chl a, chlorophyll a; Chl b, chlorophyll b; Car, carotenoid, chl a/b, chlorophyll a/chlorophyll b ratio; total chl, total chlorophyll; chl/car, chlorophyll/carotenoids ratio.

Wide variations in chl “a,” chl “b,” car and total chl content were observed (Table 3). The differences between the selected germplasm with the chl parameters were statistically significant in most cases. A highly positive significant correlation (Figure 4) was observed between chl “a” and chl “b” (*r* = 0.82), total chl to chl a (*r* = 0.98) and total chl to car ratio (*r* = 0.81), indicating that chlorophyll and carotenoids are the structural and functional components of the chloroplast, and are correlated to each other as noted earlier (67).

**TABLE 3:** Chlorophyll a, chlorophyll b, chlorophyll a/b ratio, total chlorophyll, carotenoids and chlorophyll/carotenoids ratio in the selected tea germplasm.

Selections	Chlorophyll “a” (mg g^–1^FW)	Chlorophyll “b” (mg g^–1^FW)	Chlorophyll “a/b”	Carotenoid (mg g^–1^FW)	Total chlorophyll (mg g^–1^FW)	Chlorophyll/carotenoid
TV1	2.011 ± 0.025^a^	0.821 ± 0.067^a^	2.741 ± 0.145^a^	3.080 ± 0.148^a^	2.832 ± 0.042^bc^	0.939 ± 0.037^b^c
TRA St 817	1.188 ± 0.014^c^	0.590 ± 0.019^a^	2.159 ± 0.088^a^	3.825 ± 0.055^a^	1.779 ± 0.004^e^	0.504 ± 0.018^d^
TRA St.293	1.738 ± 0.056^b^	0.626 ± 0.012^a^	2.812 ± 0.169^a^	3.125 ± 0.085^a^	2.364 ± 0.044^d^	0.826 ± 0.028^c^
TRA St.400	2.035 ± 0.030^a^	0.961 ± 0.016^a^	2.121 ± 0.005^a^	2.493 ± 0.001^a^	2.996 ± 0.046^ab^	1.211 ± 0.015^ab^
TRA 177/3	2.018 ± 0.015^a^	0.930 ± 0.011^a^	2.173 ± 0.010^a^	3.043 ± 0.223^a^	2.947 ± 0.026^ab^	1.083 ± 0.059^abc^
TRA 376/2	1.780 ± 0.029^b^	0.857 ± 0.014^a^	2.089 ± 0.064^a^	3.131 ± 0.284^a^	2.637 ± 0.015^c^	0.953 ± 0.090^b^c
TRA 376/3	1.929 ± 0.020^ab^	0.864 ± 0.009^a^	2.231 ± 0.002^a^	2.440 ± 0.025^a^	2.793 ± 0.029^bc^	1.148 ± 0.000^ab^
TRA 427/7	1.939 ± 0.014^ab^	0.870 ± 0.006^a^	2.230 ± 0.000^a^	2.743 ± 0.008^a^	2.809 ± 0.020^b^c	1.033 ± 0.000^abc^
TRA P7	1.989 ± 0.035^a^	0.913 ± 0.027^a^	2.184 ± 0.027^a^	2.335 ± 0.098^a^	2.901 ± 0.062^b^	1.249 ± 0.025^a^
TRA P8	2.077 ± 0.017^a^	1.106 ± 0.038^a^	1.893 ± 0.082^a^	3.475 ± 0.051^a^	3.183 ± 0.021^a^	0.931 ± 0.002^bc^

Values are Mean ± Standard error of the mean (SEm). Different parameters were analyzed using ANOVA to detect a significant difference between means. Means were compared using Duncan’s Multiple Range Test (DMRT) at *P* < 0.05. Mean of the same alphabets in columns are not significant. FW - Fresh weight.

Chlorophyll is the most important plant pigment since it controls the photosynthetic reaction and determines the plant’s productivity (68). The total chlorophyll content of TRA St. 817 (1.779 mg g^–1^ FW) was found at the minimum, which is very suitable for manufacturing purple tea (Table 4). Chlorophyll contributes to the “blackness of tea,” which is one of the important criteria considered in the commercial evaluation of tea (69–71). Carotenoids are important for the aroma of tea (72–76). The highest concentration of carotenoids in TRA St. 817 was 3.825 mg g^–1^ FW, followed by TRA P8 (3.475 mg g^–1^ FW), favoring the formation of more volatile flavor constituents than TV1 and other selections.

**TABLE 4:** Taster’s evaluation of promising purple tea germplasm TRA St.817.

Flush	Brightness	Briskness	Strength	Flavor/aroma	Quality
First flush	5.7	5.5	5.5	5.7	5.7
Second flush	6.0	6.2	6.2	5.8	6.3
Rain flush	4.8	5.0	5.0	4.8	4.8
Autumn flush	5.8	5.8	5.8	5.7	6.0

Mean of 3 samples/flush in a 10-point scale.

#### Correlation of chlorophyll and carotenoid pigments in the selected germplasm

The Pearson correlation coefficient between the chlorophyll and carotenoid pigments for the 10 genotypes was calculated and presented in Figure 4. Total chl content exhibited a high positive correlation with chl “a” (*r* = 0.98), chl “b” (*r* = 0.93), and chl to car ratio (*r* = 0.81), with a significant negative correlation with the car (*r* = −0.54). Similarly, the chl a/b ratio showed a significant negative correlation with total chl (*r* = −0.27) in purple tea’s selected high anthocyanin pigmented germplasm.

### Organoleptic evaluation

The rain flush showed a lighter purple color in the organoleptic examination of the prepared tea samples from TRA St. 817. The second and autumn flushes illustrate a purple color, and mellow flavor, with a brothy, mild undertone and a pleasant mouth feel (Photo plate II, Table 4). This genotype of tea has the potential to provide anthocyanin-rich special tea.

### Multiplication of TRA St. 817 germplasm

**FIGURE 5:**
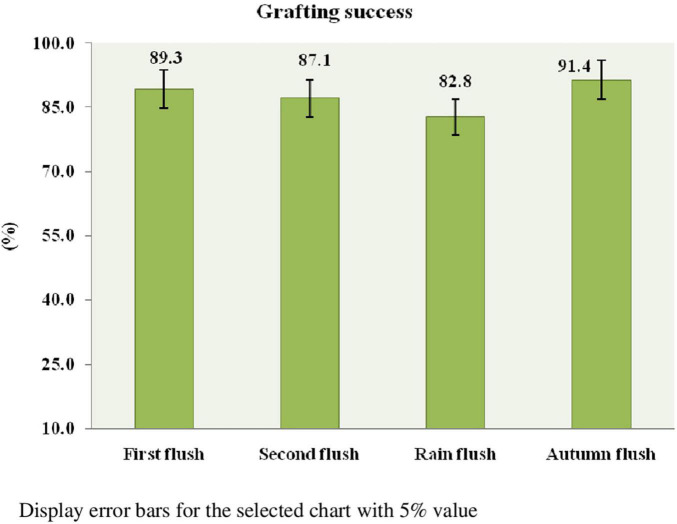
Effect of flushing on the success of grafting.

Cleft grafting was started on the first flush (March–April 2017) to the autumn flush (October–December 2017) at the Tocklai experimental plot. The study was arranged in an RBD consisting of a flushing period with three replications. A total of 15 bushes were used for cleft grafting in each flush. Each bush had a maximum of 10–12 graft unions. A total of 150–180 graft unions were established in each flush. The results show that the interaction between rootstock and scion determines graft union’s success. There is no statistically significant difference between the grafting success rates per cent in response to the flushes. This is the uniqueness of the prospective germplasm that produces a precise grafting performance among four flushes. Since the scion bud is in an ideal physiological state or dormant phase, the autumn flush had the highest success rate (91.4%). In contrast, the rain flush had the lowest success rate (82.8%) among the four flushes (Figure 5). The grafting method is illustrated in Photo plate III, and the micro-plot was established through cleft grafting (Photo plates III and IV).

## Conclusion

This study reveals that TRA St.817 is physiologically more efficient among the tea germplasm, having the highest net photosynthesis (24.7 μmol m^–2^s^–1^), water use efficiency (19.02 μmol mmol^–1^), mesophyll efficiency (ci/gs ratio: 2.54) and the highest photochemical efficiency of PSII (fv/fm: 0.754). Moreover, it is simple to multiply for mass production, with a 91.4% grafting success rate to meet future industrial demand. Regarding quality, the TRA St.817 germplasm exhibits the highest total anthocyanin pigment (4764.19 μg g^–1^ fresh leaf weight), carotenoid content (3.825 mg g^–1^ fresh leaf weight), mild caffeine (2.17%), purple colored tea liquor, mellow flavor, with a brothy, mild undertone and a pleasant mouth feel, making it a unique tea. In contrast, total chlorophyll content is significantly low (1.779 mg g^–1^ fresh weight), which is very suitable for manufacturing purple tea. For the carotenoids, the highest concentration of carotenoids in TRA St. 817 is 3.825 mg g^–1^ FW, followed by TRA P8 (3.475 mg g^–1^ FW), favoring the formation of more volatile flavor constituents. It suits for making purple tea based on physiological, biochemical, organoleptic, and multiplication performance among the nine pigmented germplasm. It has the potential to be introduced to the tea industry for the first time as novel purple tea germplasm. The outcome opens up many opportunities for commercial tea growth and diversification for growers in Northeast India in making purple tea.

## Data availability statement

The original contributions presented in this study are included in the article/Supplementary material, further inquiries can be directed to the corresponding author/s.

## Author contributions

PKP: experimental design, original draft preparation, data curation, simulation, tables, figures, and photo plates. SAS and KK: data management and interpreting. SS and RS: methodology development and HPLC analysis. BG and SKS: experimental supervision and field data generation. RKB: field trial establishment and grafting. JKS: data generation and HPLC analysis. RCG: quality analysis of made tea. KB, JY, YT, and SM: reviewing, editing, and manuscript revision. BD: micro-plot establishment and availability of resources. All authors listed have made a substantial, direct, and intellectual contribution to the work, and approved it for publication.

## Abbreviations

Pn: Net Photosynthesis
PAR: Photosynthetic Active Radiation
Tl: Leaf Temperature
Gs or gs: Stomatal Conductance
E: Transpiration
WUE: Water Use Efficiency (Pn/E)
CI or ci: Intercellular CO_2_ concentration
CA or ca: Atmospheric CO_2_ concentration
CI/CA: Carboxylation Efficiency
ME: Mesophyll Efficiency (ci/gs)
Fv/fm: Chlorophyll Fluorescence/Photochemical Efficiency of PSII
PSII: Photosystem II
TV: Tocklai Vegetative
Chl –: Chlorophyll
Car: Carotenoid
RBD: Randomized Block Design
HPLC: High-Performance Liquid Chromatography
TRA: Tea Research Association
TTRI: Tea Research Institute
EGC: epigallocatechin + C-catechin
EC: epicatechin
EGCG: epigallocatechin gallate
EGC: epicatechin gallate.
