# Supplementary material for: Physiological and biochemical evaluation of high anthocyanin pigmented tea (Camellia sinensis L. O. Kuntze) germplasm for purple tea production
Source: Front Nutr. 2022 Aug 31;9:990529. doi: 10.3389/fnut.2022.990529 (PMC9471081; doi:10.3389/fnut.2022.990529)
Supplement: Supplementary file 1 [file Data_Sheet_1.pdf]

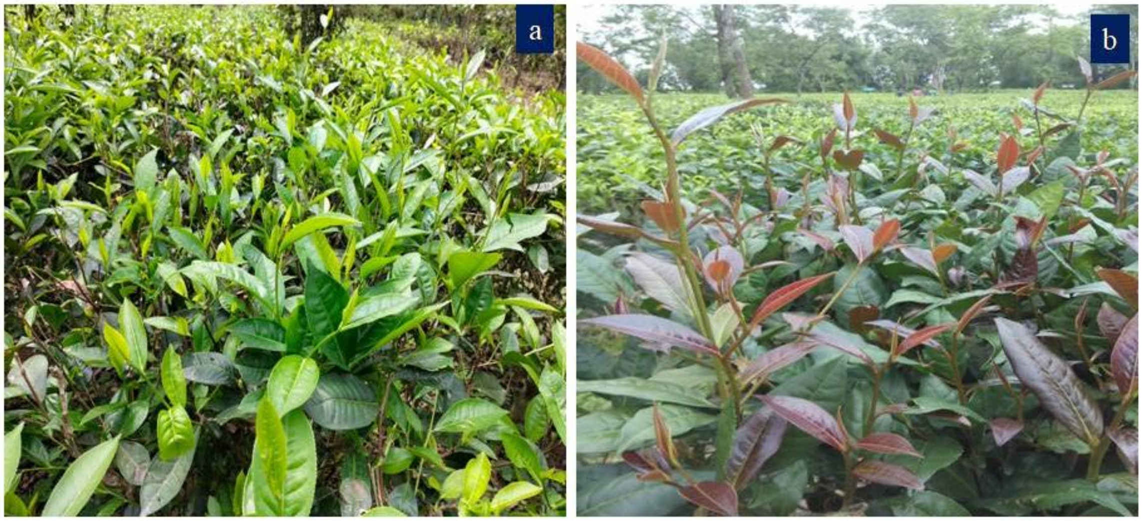

Photo Plate-I

Photo plate I (a) Green coloured tea plants (TV1) (b) Purple coloured tea plants

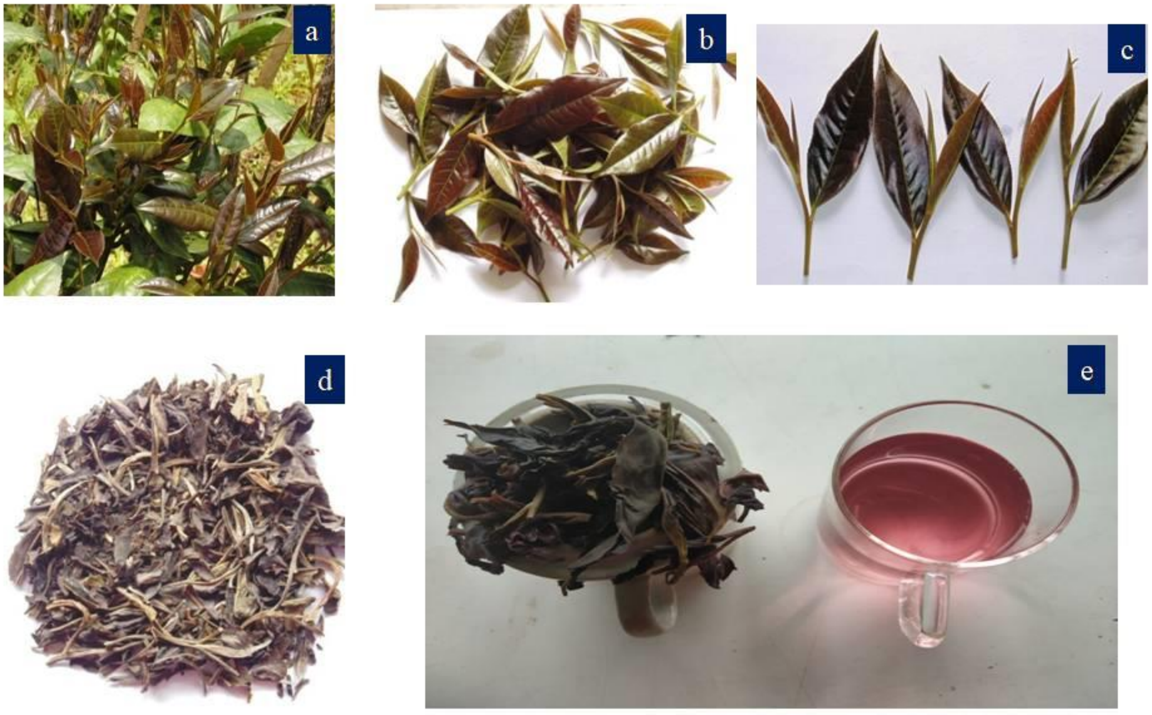

Photo plate II Promising purple tea germplasm TRA St. 817 (a) Purple tea bush (b) Plucked leaves (c) Tea shoot - two leaf and a bud (d) Made tea (e) Infused leaf and tea liquor

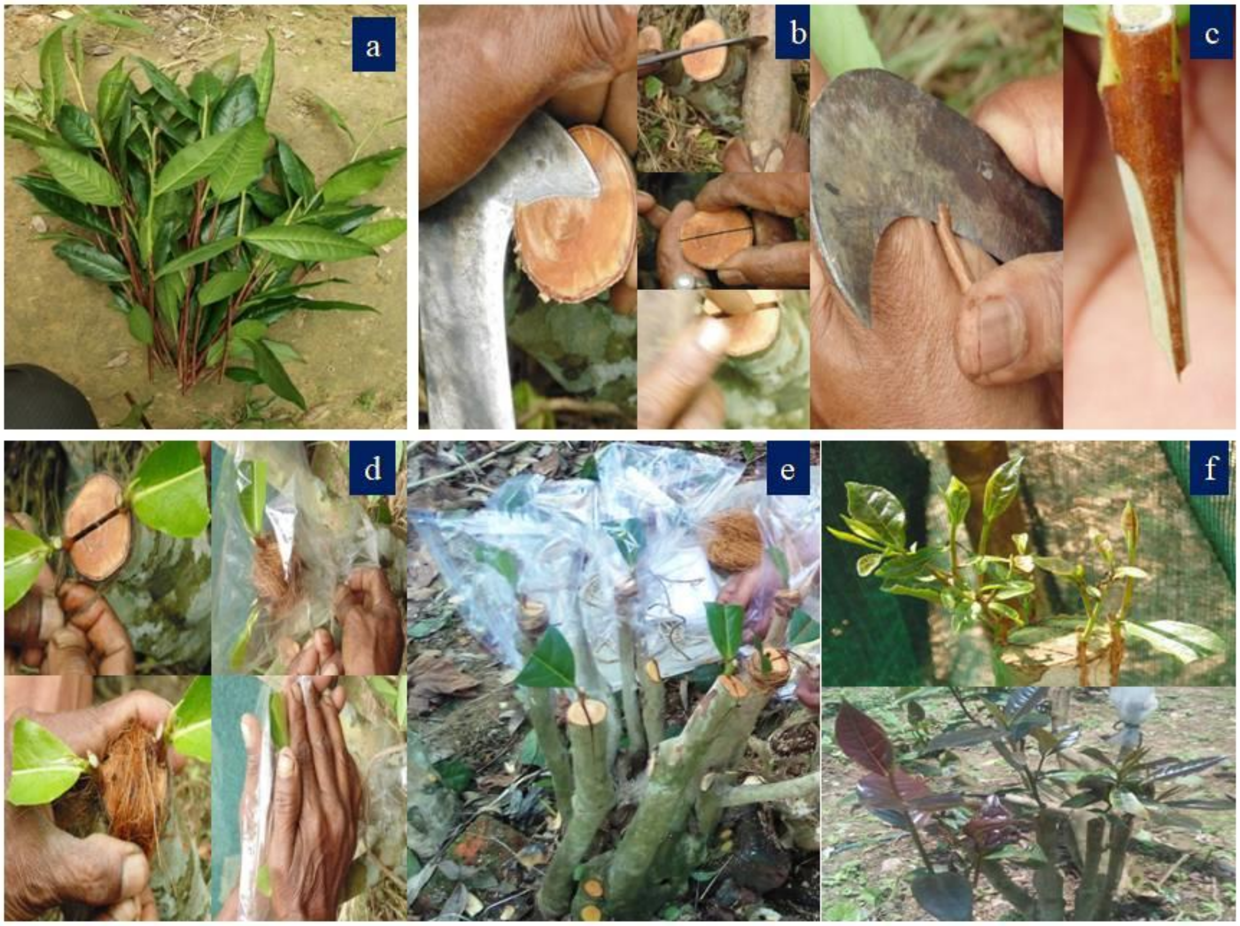

Photo plate III Illustration of cleft grafting (a) Semi-hard parts of the primaries collected from the raised bush for scion (b) Stock-cut the selected branches horizontally, making the cut surface smooth using a sharp pruning knife and making a cleft on the clean-cut stock branch at the centre with the help of a sharp pruning knife. For widening the cleft, a bamboo wedge was inserted at the center (c) Scion-give a slashing cut in the inter-node, 0.5-1.0 cm below the lower leaf, from both sides to form a tapering end (d) Inserting two prepared scions at the two ends of the cleft made on the stock branch in such a way that the cambium layers of both scion and stock closely contact and remain in line. Then the bamboo wedge was slowly and carefully removed from the cleft, and the entire cleft was tied with jute thread. The exposed grafting surface of the stock branch was covered with moist coconut coir after squeezing out the excess water and tied with jute thread. The entire graft was carefully covered with a polythene bag not to touch the grafted scions, and the bag was tied below the stock branch. The overhead shade was required and was open towards the north for successful scion stock union (e) Removal of polythene bags (f) Hardening of the newly emerging shoots

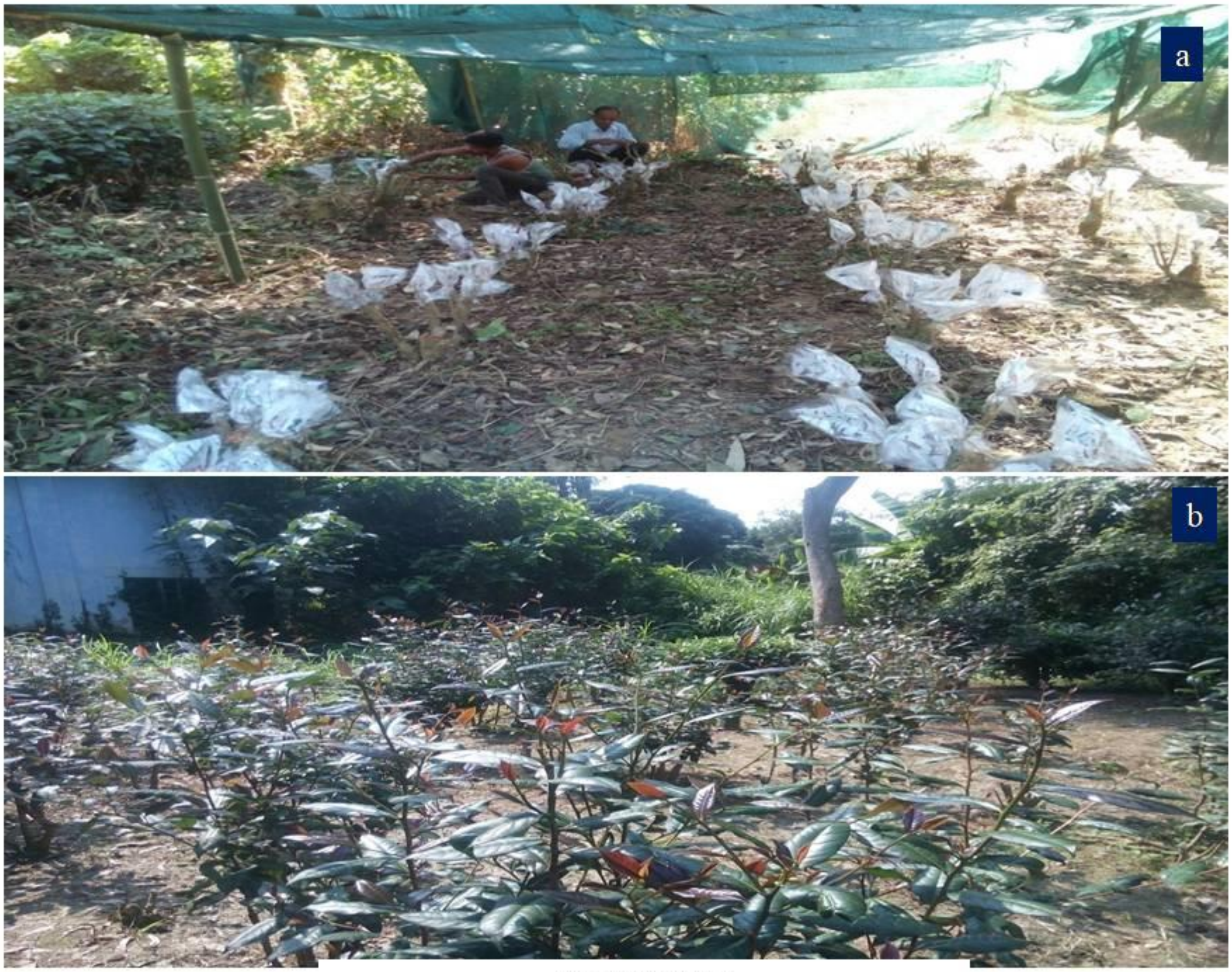

IV Photo plate IV Multiplication of TRA St. 817 (a) Cleft grafting initiated (b) Established plot
