# Supplementary figures and images for: Physiological and biochemical evaluation of high anthocyanin pigmented tea (Camellia sinensis L. O. Kuntze) germplasm for purple tea production
Source: Front Nutr. 2022 Aug 31;9:990529. doi: 10.3389/fnut.2022.990529 (PMC9471081; doi:10.3389/fnut.2022.990529)

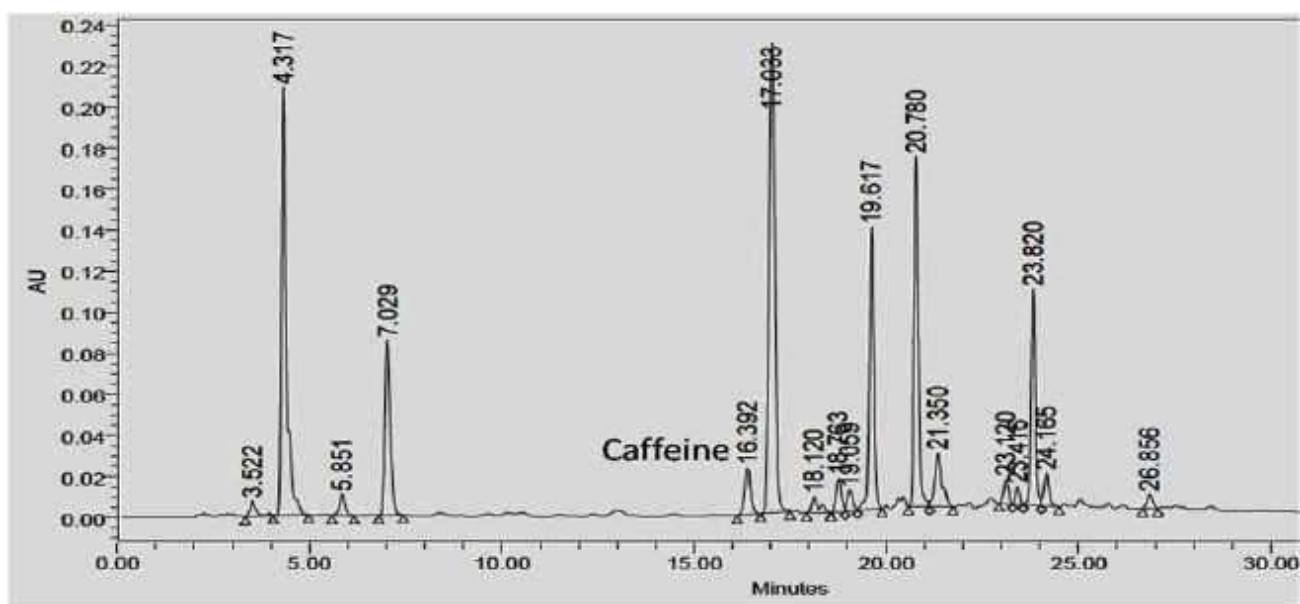

**Supplementary figure 1:** HPLC chromatogram of the tea made from TRA St.817.

Supplement: Supplementary file 2 [file Data_Sheet_2.pdf]
